# Congenital Colonic Stenosis in a Preterm Infant: A Diagnostic Trap and the Potential Masking Effect of a Liquid Diet

**DOI:** 10.3390/children13091168

**Published:** 2026-08-29

**Authors:** Chee-Chee Koh, Pi-Feng Chang

**Affiliations:** 1 Division of Pediatric Surgery, Department of Surgery, Far Eastern Memorial Hospital, No. 21, Section 2, Nanya South Road, Banqiao District, New Taipei City 220056, Taiwan; 2 Department of Pediatrics, Far Eastern Memorial Hospital, No. 21, Section 2, Nanya South Road, Banqiao District, New Taipei City 220056, Taiwan; honeybee7689@gmail.com

**Keywords:** colonic stenosis, contrast enema, infant, intestinal obstruction, premature infant, diagnostic error

## Abstract

**Highlights:**

**What are the main findings?**

- A dietary masking effect delayed presentation: liquid stool passed through the critically narrowed lumen, maintaining a reassuring picture until solid foods increased stool viscosity and triggered acute obstruction.
- An anchoring error created a diagnostic trap: because the infant thrived on loose stools, a reproducible contrast cutoff was dismissed as a procedural artifact, a bias reinforced when normal rectal biopsy excluded Hirschsprung disease.

**What are the implications of the main findings?**

- Reassuring growth and loose stools reflect stool viscosity relative to the lumen, not absence of a lesion; new obstructive symptoms after solid-food introduction warrant reconsidering a structural cause.
- A negative workup for the likeliest differential does not validate dismissing an unexplained imaging finding; reproducible structural abnormalities warrant investigation on their own merits.

**Abstract:**

**Background:** Congenital colonic stenosis (CCS) is a rare cause of neonatal bowel obstruction due to a focal luminal narrowing. We report a premature infant in whom a high-grade CCS remained clinically compensated for months during exclusively liquid feeding. **Case Presentation:** An 8-month-old corrected-age female infant (born at 28^+5^ weeks’ gestation; body weight (BW): 1095 g) presented to the Emergency Department with acute abdominal distension, bilious emesis, and obstipation, despite a history of steady weight gain and chronic mild abdominal distension. A neonatal contrast enema had previously shown an abrupt cutoff suggestive of obstruction, but this was attributed to a procedural artifact given her tolerance of feeds and passage of loose stools; a rectal suction biopsy excluded Hirschsprung disease. Within four days of introducing complementary solid foods at 8 months corrected age, her compensation failed and complete functional obstruction developed. A repeat contrast enema confirmed a short-segment, annular stenosis of the distal descending colon. Conservative management with a liquid diet failed, and definitive surgical resection with primary anastomosis was performed approximately one month later, achieving immediate symptom resolution and long-term catch-up growth. **Conclusions:** Reassuring weight gain and liquid stool passage can create a diagnostic trap, masking a high-grade colonic structural obstruction. Clinicians must not disregard reproducible focal anomalies on neonatal contrast imaging based solely on clinical plausibility.

## 1. Introduction

Neonatal distal bowel obstruction can be broadly categorized into mechanical and functional etiologies. Mechanical obstruction arises from physical blockages, anatomical discontinuities, or abnormal luminal contents that impede the passage of stool, with representative congenital conditions including meconium plug syndrome, meconium ileus, small left colon syndrome, and distal intestinal atresia or stenosis [1]. Conversely, functional obstruction occurs when the structural continuity of the bowel remains intact, but defective neuromuscular transmission compromises effective peristalsis. The most prevalent of these functional disorders is Hirschsprung disease, whereas conditions such as intestinal neuronal dysplasia or pseudo-obstruction remain exceedingly rare [1,2]. Additionally, acquired etiologies, such as post-necrotizing enterocolitis (NEC) intestinal strictures, can induce distal bowel stenosis, particularly in preterm neonates [3,4].

Congenital colonic stenosis (CCS) represents a rare variant of lower gastrointestinal obstruction [5,6]. While its exact pathogenesis remains debated, proposed mechanisms include fetal mesenteric vascular accidents, intrauterine ischemic insults, and incomplete intestinal recanalization [4,5]. Due to incomplete luminal narrowing, its clinical presentation is highly variable, traditionally ranging from acute neonatal ileus or early childhood failure to thrive [7,8] to insidious chronic constipation that may closely masquerade as Hirschsprung disease [6,9]. Consequently, the preoperative diagnosis of CCS presents a significant challenge for both clinicians and radiologists [5,9]. Although a contrast enema is considered the essential diagnostic modality to delineate the transition from a small-caliber distal colon to a dilated proximal colon, its findings can be inconclusive or misleading in clinical practice [1,10].

We present a case of a preterm female infant with persistent bowel dilatation who paradoxically exhibited none of these clinical indicators; she presented with no bowel obstruction, no failure to thrive, and no history of constipation. Because the infant maintained steady, age-appropriate weight gain and passed regular fluid stools, the pediatrician incorrectly attributed the early abnormal obstructive findings on her neonatal contrast enema to a procedural artifact. This report examines this critical diagnostic pitfall, demonstrating how an infant’s liquid-exclusive diet may clinically mask a severe, pinpoint descending colon stenosis, delaying definitive diagnosis until the introduction of complementary solid foods alters stool consistency and abruptly precipitates an acute functional bowel obstruction.

## 2. Case Report

An 8-month–2-day corrected-age (CA) female infant (postnatal day 327), born prematurely at 28^+5^ weeks with a birth weight of 1095 g, presented with a 3-day history of progressive abdominal distention, bilious emesis, and obstipation. Her acute symptoms began within four days of introducing complementary solid foods.

**Figure 1:**
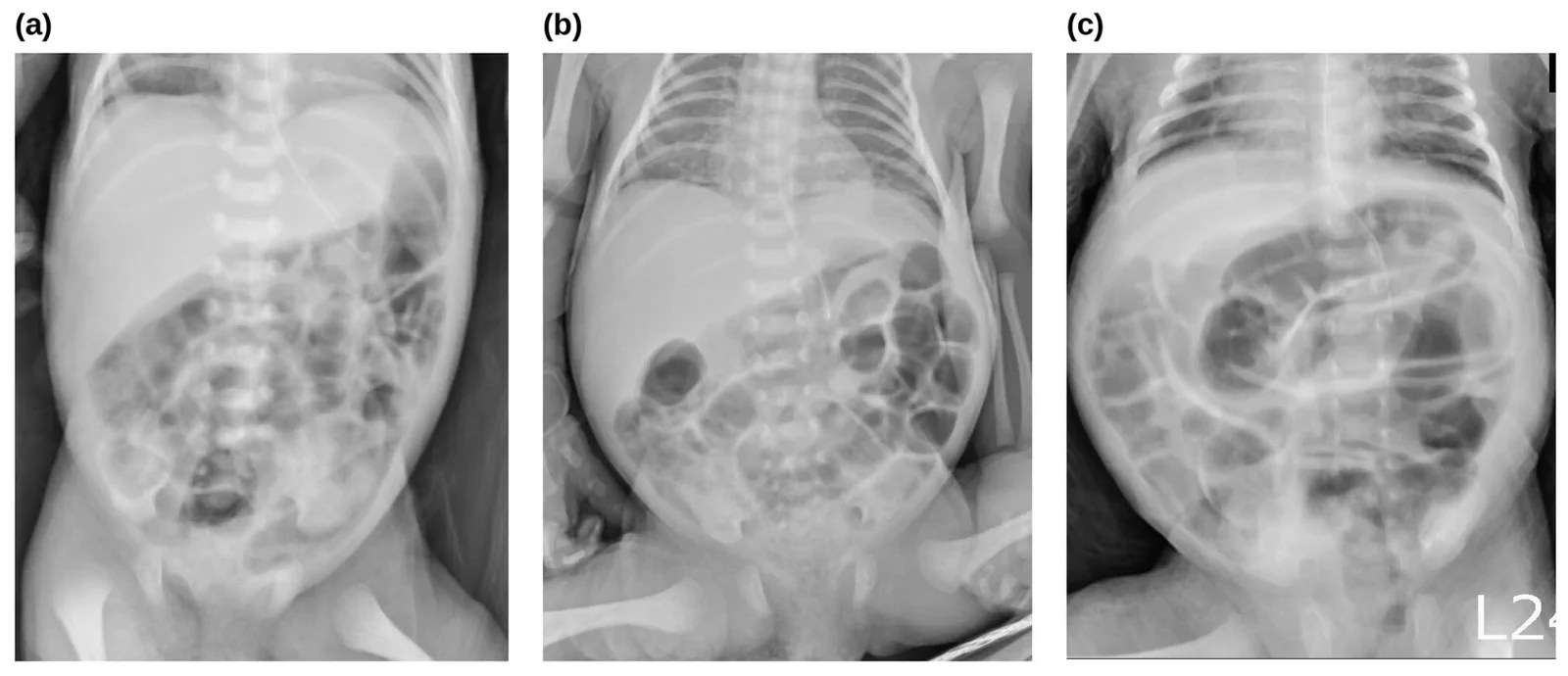
Serial anterior–posterior abdominal radiographic views demonstrating persistent, progressive generalized bowel dilatation since day 10. Views were performed at chronological age and PMA accordingly: (**a**) Day 10 (PMA 30 weeks), (**b**) Day 17 (PMA 31 weeks), and (**c**) Day 62 (PMA 37^+3^ weeks). PMA: Postmenstrual age.

Her neonatal course was complicated by persistent bowel dilatation first noted on day 10 (postmenstrual age [PMA]: 30 weeks), which progressed on serial radiographs (Figure 1) without clinical evidence of complete obstruction. History was negative for infectious enteritis or necrotizing enterocolitis. Despite distention, she tolerated enteral breast milk and formula, passing loose stools 2 to 10 times daily.

**Figure 2:**
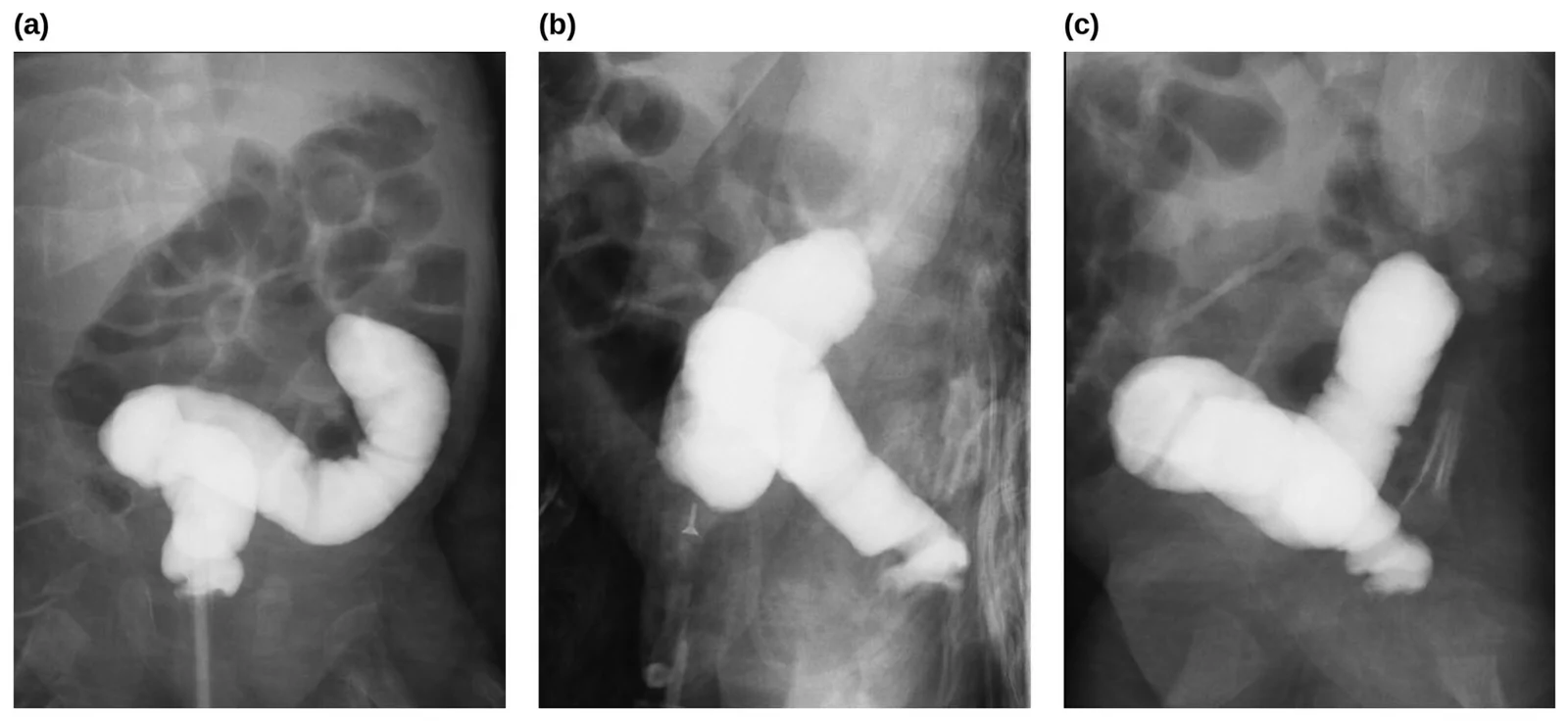
The first contrast enema was performed on postnatal day 68 (PMA 38^+2^ weeks; BW 2190 g). (**a**) Anteroposterior, (**b**) lateral, and (**c**) oblique views demonstrating an abrupt retrograde cutoff of the contrast medium within the distal descending colon, suggesting distal colon obstruction. PMA: Postmenstrual age.

A fluoroscopic contrast enema on postnatal day 68 (body weight (BW) 2190 g) for protracted abdominal distention showed an abrupt cutoff in the distal descending colon (Figure 2). A rectal suction biopsy on day 69 confirmed normal ganglion cells, ruling out Hirschsprung’s disease. Given that the patient tolerated full enteral volumes and demonstrated appropriate weight gain, the abnormal contrast enema findings were clinically discounted at that time and attributed to a technical artifact or transient functional causes. The patient was discharged on day 80 (PMA: 40^0^ weeks; BW: 2430 g).

At home, an exclusive liquid diet maintained steady weight gain, masking the underlying anomaly. Routine well-child visits noted occasional mild abdominal distension, but without vomiting, obstipation, or growth faltering; no repeat imaging was performed.

At 8 months corrected age (postnatal day 327, BW: 7800 g), introducing complementary foods precipitated acute obstipation, bilious emesis, and severe abdominal distention. In the emergency department, venous blood analysis reflected a primary respiratory alkalosis due to tachypnea from pain, with a superimposed mild metabolic acidosis from dehydration. Intravenous fluid resuscitation resolved the acute signs, allowing oral milk intake to resume by hospitalization day 3.

**Figure 3:**
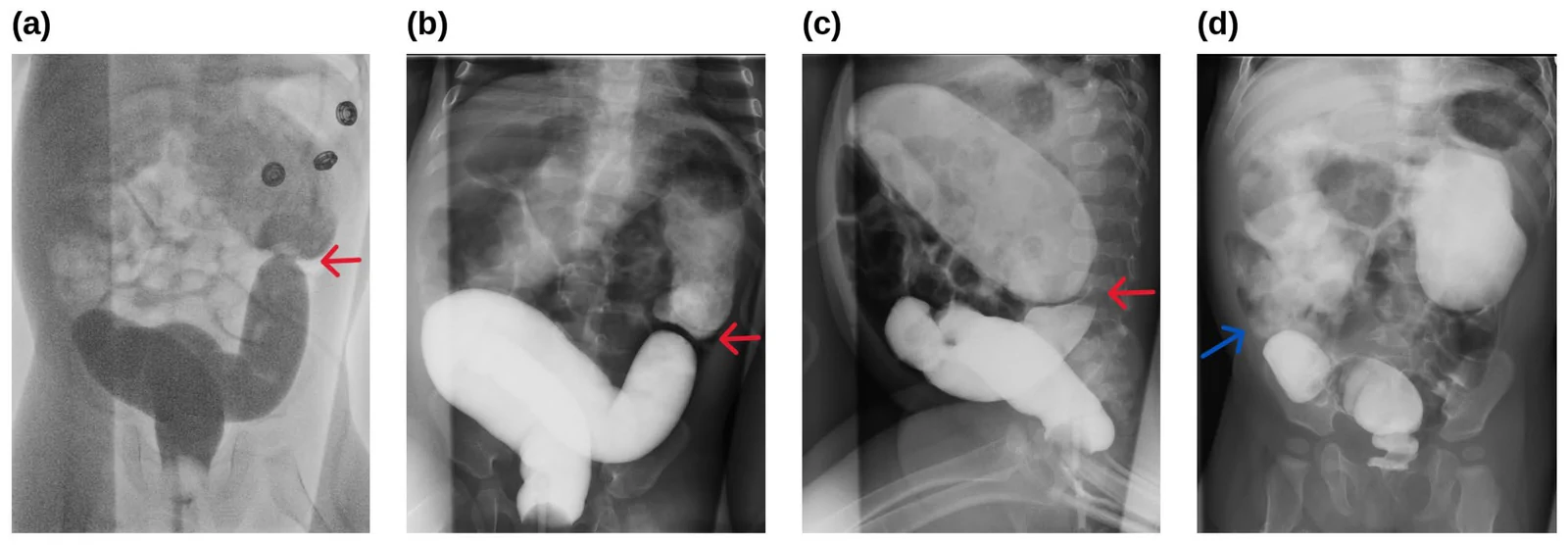
Repeat fluoroscopic contrast enema performed at 8 months of corrected age. (**a**,**b**) Anteroposterior and (**c**) lateral radiographic views revealing the passage of contrast through a localized, annular constriction (red arrows) within the descending colon. (**d**) Post-evacuation view illustrating retrograde migration of the contrast medium through the stenotic segment and the transverse and ascending colon to the cecum. The cecum is positioned within the right iliac fossa (blue arrow); no synchronous stenosis was demonstrated.

A repeat contrast enema encountered substantial retrograde resistance, demonstrating a persistent focal luminal narrowing and annular constriction in the descending colon (Figure 3a–c). The post-evacuation view showed retrograde migration of contrast through the stenotic segment into the transverse colon, ascending colon, and cecum, with no synchronous stenosis (Figure 3d). These findings established a definitive, high-grade focal mechanical stenosis, structurally correlating with the severe obstructive symptoms triggered by complementary solid foods.

**Figure 4:**
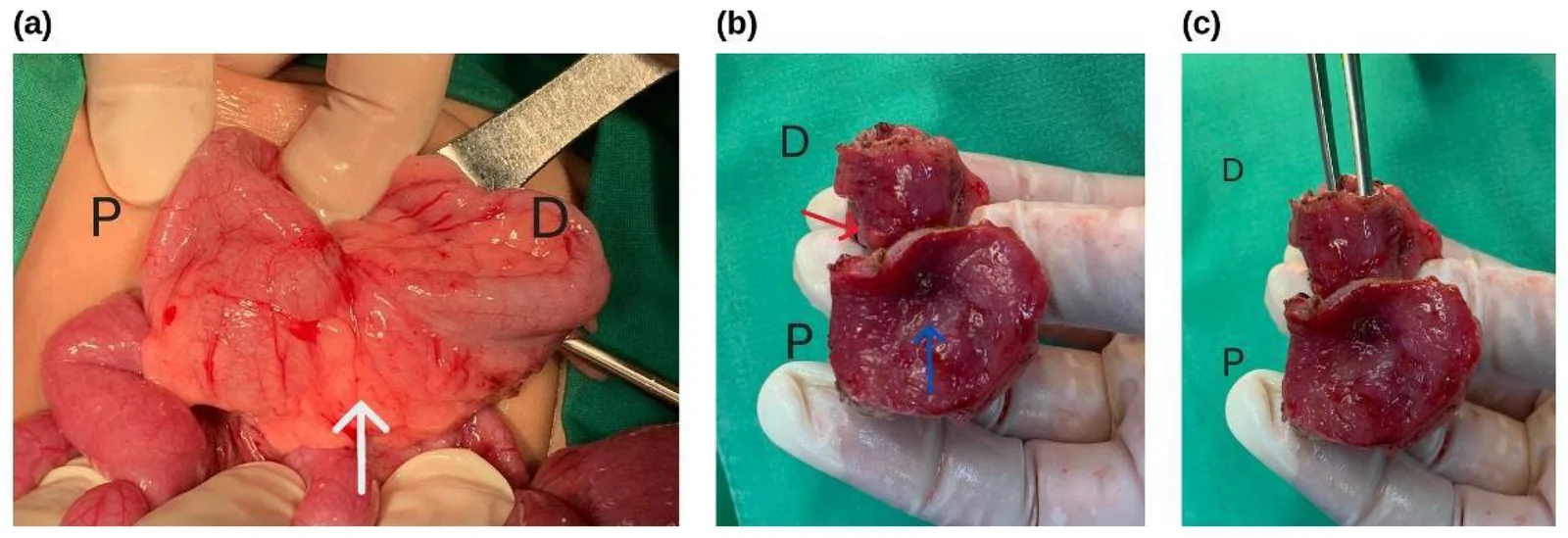
Intraoperative findings of the colonic stricture. (**a**) Intraoperative photograph showing colonic stenosis obscured by dense mesenteric adhesions and fibrofatty proliferation (white arrow). (**b**,**c**) Resected specimen demonstrating advanced luminal narrowing. (**b**) The residual lumen is reduced to a pinpoint stricture (blue arrow), presenting externally as a distinct, focal annular constriction (red arrow). (**c**) Severe luminal stenosis preventing passage of standard surgical forceps. D: distal; P: proximal.

The family initially opted for reverting to a liquid diet, but due to weight loss and food intolerance, they elected surgery one month later. At 9 months CA, an exploratory laparotomy revealed a very short-segment stenosis at the distal descending colon, circumferentially surrounded by dense mesenteric adhesions and fibrofatty proliferation. Segmental resection was performed 2 cm proximal and distal to the stenotic segment, identifying a critically narrowed lumen (Figure 4). A primary end-to-end anastomosis was successfully completed.

Gross examination of the specimen revealed severe concentric narrowing with a residual lumen of approximately 2 mm (Figure 4b,c). Histopathology showed normal ganglion cells and prominent mural fibrosis localized to the stenotic area, with no evidence of active inflammation. These findings definitively excluded Hirschsprung’s disease, establishing a conclusive diagnosis of focal colonic stenosis.

Postoperatively, the patient tolerated dietary advancement and was discharged on day 7. By 6 months post-surgery (15 months CA), she tolerated solid foods and passed normal stools. Catch-up growth was significant: body weight reached 10.0 kg at 19 months CA. At last follow-up (60 months CA; 5 years), she weighed 17.0 kg (z-score −0.5) and measured 108 cm (z-score −0.3). She passed one stool daily without laxatives, was continent, and had no anastomotic strictures or recurrent obstructions.

**Figure 5:**
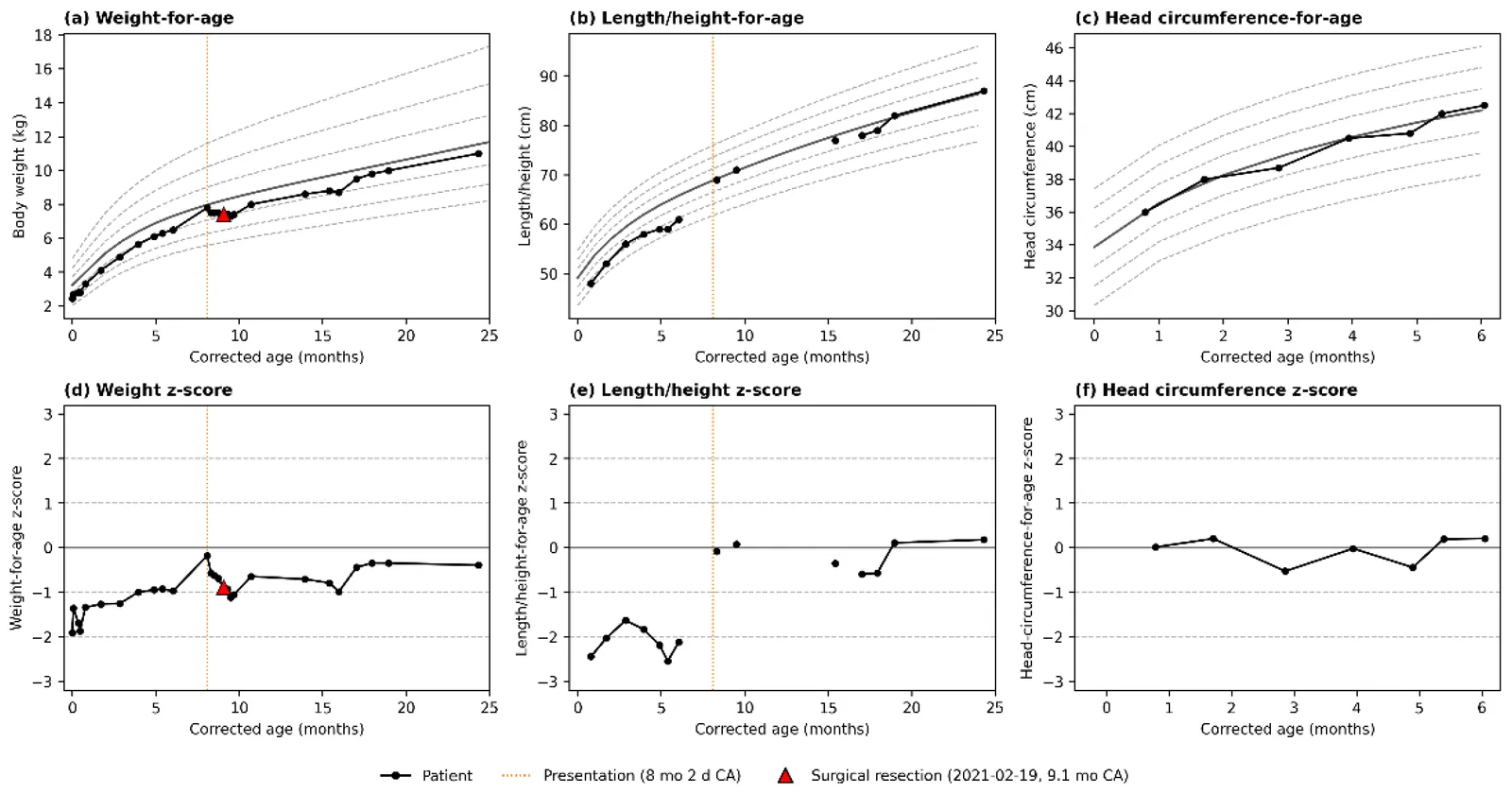
WHO Child Growth Standards trajectories. Trajectories are corrected for prematurity (28^+5^ weeks). (**a**–**c**) Absolute weight, length/height, and head circumference plotted against WHO reference curves (median [solid gray] and ±1, ±2, ±3 SD [dashed gray]). (**d**–**f**) Corresponding z-scores, with reference lines at 0, ±1 SD, and ±2 SD. Orange dotted line: presentation with acute obstruction (8 months 2 days CA); red triangle: surgical resection (9.1 months CA). Head circumference recording stops at 6 months CA per routine schedule; panels (**c**,**f**) are truncated accordingly. Z-scores are derived by the LMS method using WHO reference parameters for girls, with monthly LMS values linearly interpolated to each measurement’s exact corrected age in days.

Objective anthropometric assessment confirmed masked obstruction. The weight-for-age z-score was −1.9 at discharge, rising during exclusive liquid feeding to −1.0 at 6 months CA and −0.2 at presentation (8 months 2 days CA). The z-score slightly decreased to −0.9 at surgery (9.1 months CA; weight 7.4 kg), then progressively recovered to −0.3 to −0.4 by 19–24 months CA (Figure 5a,d).

Length and head circumference tracks revealed a pattern hidden by weight alone. Pre-operative length-for-age z-scores remained substantially lower than weight-for-age (−2.1 to −2.4, near stunting thresholds), normalizing to +0.1 by 19–24 months CA after surgery (Figure 5b,e). This dissociation indicates that weight gain—the driver of diagnostic masking—was preserved more than linear growth. Conversely, head circumference-for-age z-scores remained normal from term to 6 months CA (−0.5 to +0.2), demonstrating brain-sparing growth despite linear growth lagging (Figure 5c,f).

## 3. Discussion

Congenital colonic stenosis (CCS) is a rare gastrointestinal anomaly with no incidence established specific to the stenotic subtype; the closest available population-based estimate for combined colonic atresia and stenosis is approximately 0.15 per 10,000 live births [11]. Unlike colonic atresia, which presents as complete luminal occlusion requiring immediate neonatal diagnosis [12], CCS retains a minimal patent lumen that permits variable, compensated transit of luminal contents—the anatomical basis for the delayed, intermittent presentations (failure to thrive, colicky pain, chronic constipation) reported in the literature [4,6,7,8,9,10,13,14,15], and for the masking phenomenon this case illustrates.

Crucially, this case highlights a dietary “masking effect” inherent to infantile partial intestinal obstruction. During exclusive milk-feeding, the low viscosity of breast milk or formula allowed liquid stool to pass through the stricture, maintaining regular bowel movements and age-appropriate weight gain—reflected in the near-normalization of her weight-for-age z-score by presentation (Figure 5a,d). Consequently, a high-grade mechanical obstruction remained unrecognized until 8 months corrected age (CA), when introducing complementary solid foods produced bulkier, formed stools that failed to pass through the critically narrowed lumen, precipitating progressive obstruction. Although the family temporarily reverted to a liquid diet, the infant lost 400 g and developed emesis and obstipation 2 days pre-operatively, indicating the obstruction had progressed beyond what dietary modification could relieve. While causation cannot be definitively established from a single case, other contributing factors may include progressive narrowing of the stenotic segment, altered colonic motility, age-related increases in stool volume, and chronic proximal dilatation with impaired propulsive efficiency.

Intraoperative findings confirmed a localized descending colonic stenosis enveloped by dense mesenteric adhesions and reactive fibrofatty proliferation, without evidence of acute inflammatory change; histology showed severe concentric luminal narrowing with mural fibrosis strictly localized to the stricture site. These findings alone could not distinguish a congenital from an acquired etiology, but the neonatal record argued against the latter: the infant had no necrotizing enterocolitis, perforation, sepsis, prolonged hypotension, vasopressor exposure, or prior abdominal surgery, and achieved full enteral autonomy by postnatal day 33 without parenteral support. The dense adhesions and fibrofatty proliferation, in an abdomen with no history of surgery or inflammatory insult, are more consistent with a localized antenatal mesenteric vascular event than a simple failure of recanalization, reflecting fetal healing after a prenatal ischemic insult rather than a postnatal inflammatory process [3], though a contribution from slow tissue remodeling cannot be excluded, since histology reflects only late-stage pathology at resection. Taken together, this unremarkable neonatal course and the histological and radiological findings establish the diagnosis of congenital colonic stenosis. Unlike colonic atresia, in which associated cardiac, renal, and skeletal anomalies are a well-recognized concern [16], isolated CCS has not been as consistently linked to extraintestinal malformations; formal anomaly screening was therefore not pursued, and none were apparent on clinical examination throughout follow-up.

A fluoroscopic contrast enema is the essential modality for distinguishing colonic stenosis from atresia [9,10]: atresia typically shows a microcolon with an absolute, abrupt cutoff, whereas stenosis manifests as a concentric or annular filling defect [10,17], distinguishing it also from the non-relaxing, cone-shaped transition zone of Hirschsprung disease [6,9]. Diagnostic workup for CCS can be genuinely inconclusive, since retrograde contrast propagation depends on catheter positioning, hydrostatic pressure, contrast viscosity, angle of entry, bowel spasm, and stenosis morphology [1,5,10]. On retrospective review, our patient’s initial neonatal enema showed this distinct, identifiable cutoff sign, but it was overlooked for two reasons: the infant’s low weight (2190 g) led to an assumption that hydrostatic pressure had been deliberately minimized, artificially limiting contrast propagation, and the finding directly contradicted her reassuring clinical picture of tolerated feeds and regular loose stools—together leading the study to be misattributed to an incomplete procedure rather than true pathology.

Importantly, the neonatal enema was correctly interpreted by the radiology team at the time, with the report explicitly describing an abrupt retrograde cutoff suspicious for a distal obstructive lesion. The diagnostic failure therefore originated not from image interpretation, but from subsequent clinical decision-making. The treating team weighed the radiological finding against the infant’s reassuring clinical trajectory, ultimately dismissing it as a technical artifact rather than pursuing it as a structural lesion. This illustrates a clinical anchoring error driven by discordance between an accurate radiological report and a falsely reassuring clinical picture. It underscores that a reproducible, well-described imaging abnormality warrants investigation on its own merits and should not be dismissed based on clinical plausibility alone. Ultimately, the failure of retrograde contrast passage did not imply complete anatomical occlusion, as a minute residual lumen remained patent throughout.

Rectal suction biopsy, performed because Hirschsprung disease is the most common mimic of a fixed structural lesion in this age group, showed normal ganglion cells—excluding Hirschsprung disease, but not accounting for the separate structural abnormality already seen on the contrast study. In retrospect, this negative biopsy was itself an anchoring error: reassurance about the excluded differential diagnosis was mistaken for reassurance about the unrelated imaging finding, rather than being recognized as an unresolved discrepancy.

When persistent symptoms or unexplained growth faltering remain unresolved, a repeat contrast enema is warranted [8,10]. In this case, a high index of suspicion prompted repeat fluoroscopy, which demonstrated the localized annular constriction and enabled definitive surgical planning. Because CCS can present with multiple synchronous stenoses, comprehensive pan-colonic evaluation is important [10,17]; here, operative inspection of the entire colon was omitted because real-time fluoroscopy had already confirmed continuous retrograde contrast migration from rectum to cecum, excluding synchronous lesions.

Surgical management must be tailored to the patient’s clinical status, lesion location, and luminal diameter discrepancy [5,13]; primary end-to-end anastomosis is generally safe when the proximal-to-distal ratio is 3:1 or less [5,13]. In our patient, this ratio was approximately 2:1 on both preoperative enema and intraoperative confirmation, reconciled with a minor longitudinal spatulation of the distal colonic end to permit a tension-free primary anastomosis. Following an uneventful postoperative course, the infant exhibited substantial catch-up growth. A staged approach—initial divided stoma followed by delayed anastomosis—remains preferable when nutritional status, sepsis, or comorbidities are unfavorable [5].

## 4. Conclusions

In infants on exclusive liquid diets, high-grade colonic stenosis can remain compensated, preserving normal defecation and weight gain. Reassuring growth and loose stools must not override reproducible focal abnormalities on contrast imaging, even if Hirschsprung disease is biopsy-excluded. Decompensation can occur during transition to complementary solids; this change in stool consistency—rather than lesion progression—triggers acute obstruction and warrants re-evaluation. Timely surgical correction achieves sustained long-term normalization of growth parameters.

## Data Availability

The original contributions presented in this study are included in the article. Further inquiries can be directed to the corresponding author.

## Abbreviations

The following abbreviations are used in this manuscript:

CCS	Congenital colonic stenosis
CA	Corrected age
BW	Body weight
PMA	Postmenstrual age
g	Gram
SD	Standard deviation
WHO	World Health Organization
LMS	Lambda–Mu–Sigma
